# Tai Chi for Improving Chronic Primary Musculoskeletal Pain: Protocol for a Systematic Review

**DOI:** 10.1155/2021/9932336

**Published:** 2021-06-21

**Authors:** Shan Gao, Xixiu Ni, Zhenxi He, Yanan Wang, Mingsheng Sun, Lu Liu, Yang Yu, Qing Liu, Xingyu Chen, Jianwei Wu, Ling Zhao

**Affiliations:** ^1^Acupuncture and Tuina School, Chengdu University of Traditional Chinese Medicine, Chengdu, China; ^2^Clinical Research Center for Acupuncture and Moxibustion in Sichuan Province, Chengdu, China; ^3^Chinese Classics School, Chengdu University of Traditional Chinese Medicine, Chengdu, China

## Abstract

*Background*. Chronic primary musculoskeletal pain (CPMP) is a major health problem that has physical and psychological impacts as well as an associated economic burden. Currently, pharmacological treatment remains unsatisfactory because of side effects and potential misuse. Therefore, nonpharmacological approaches for pain are being actively explored, and Tai Chi has attracted increased attention as a therapy for pain. Although clinical trials have shown that Tai Chi may be effective in treating CPMP, no systematic review has clarified its effectiveness and safety. The objective of this systematic review is to assess the effect and safety of Tai Chi for patients suffering from CPMP. *Methods and Analysis*. We will search relevant electronic databases from inception to May 31, 2021: PubMed, EMBASE, Cochrane Central Register of Controlled Trials, China National Knowledge Infrastructure, the Wanfang database, the Chongqing VIP database, and China Biology Medicine Disc. Studies comparing the use of Tai Chi with other managements for CPMP patients will be included. Our review will include studies that measured change in pain intensity as the primary outcome using patient-reported ratings (visual analog scale or numerical rating scale). Hospital Anxiety and Depression Scale scores, SF-36 Health Survey scores, Pittsburgh Sleep Quality Index scores, and adverse effects will be explored as secondary outcomes. The risk of bias and the reporting quality of included studies will be evaluated using the Cochrane Collaboration risk-of-bias assessment method. The data will be analyzed using RevMan v5.3 software. *Study Registration*. This study protocol was registered on PROSPERO. The registration number for this protocol is CRD42020165048.

## 1. Introduction

Chronic primary musculoskeletal pain (CPMP), including chronic primary neck pain, chronic primary thoracic pain, chronic primary low back pain, and chronic primary limb pain, is the most common chronic pain and represents considerable global health and socioeconomic burden [1, 2]. For example, one in four adults across Europe experiences musculoskeletal disorders [1]. In 2017, low back pain was ranked the leading cause of disability (years lived with disability), with the prevalence of neck pain and other musculoskeletal conditions also being high [3]. Patients with chronic pain generally suffer progressive autonomic symptoms, such as mental stress, anxiety, and sleep disorders [4]. Physical and mental stress also affect the quality of life, daily activities, and employment, which lead to a considerable socioeconomic burden [5].

Although CPMP has an extensive impact, effective management of CPMP remains lacking. Also leading to physical dysfunction, CPMP induces prolonged sleep deprivation and negative emotions such as anxiety and depression, which in turn exacerbate the susceptivity to pain and decline in the quality of life [6–8]. Pain management is also complicated by sleep, mental, and physical problems. A holistic approach for treating pain should consider multiple factors including biological, physical, psychological, social, and other associated factors [9, 10]. Unfortunately, clinicians generally seek to manage pain through biomedical approaches and do not comprehensively consider the psychological and physical factors associated with CPMP [6]. Pharmacological treatment focused on pathology results in insubstantial improvement of accompanying negative emotions and health-related quality of life [11, 12]. Besides, there is some controversy regarding the use of medication for pain because of side effects [13] and potential misuse [14, 15]. Therefore, it is essential to explore and develop effective and nonpharmacological treatments for CPMP.

As a significant component of nonpharmacological treatment, Tai Chi is playing an increasingly important role in chronic disease management. Tai Chi has shown important contributions in the management of various common diseases, such as chronic obstructive pulmonary disease [16, 17], heart failure [11, 18], cognitive impairment [13, 19, 20], and insomnia [21, 22], which appear to be comorbid health conditions for chronic pain [23, 24]. Previous trials also showed that Tai Chi had benefits for chronic pain [25–27]. Specifically, it has been demonstrated that Tai Chi is an enjoyable, easy-to-learn exercise that induces relaxation and pain relief through increasing endogenous opioid signals [28, 29]. Tai Chi also contributes to improvement in the physical performance [30, 31] and health-related quality of life [25, 31, 32] and has beneficial effects on psychological well-being and sleep quality [33]. As the most common type of chronic pain, it is likely that CPMP will be a condition alleviated by Tai Chi.

After a preliminary search of relevant databases, we found an increasing number of studies on Tai Chi for CPMP. However, the evaluation of the efficacy of Tai Chi varied among clinical trials. A series of systematic reviews that aimed at detecting the effect of Tai Chi for chronic musculoskeletal pain (CMP) have been published [34, 35]. Unfortunately, some common types of CMP such as chronic primary low back pain and chronic primary neck pain were not considered in these reviews. Therefore, the objective of this systematic review is to evaluate the effect and safety of Tai Chi for patients suffering from CPMP.

## 2. Methods and Analysis

### 2.1. Study Registration and Report

This study protocol was registered on PROSPERO. The registration number for this protocol is CRD42020165048. The protocol report followed the Preferred Reporting Items for Systematic Reviews and Meta-Analysis Protocols (PRISMA-P) guidelines. In addition, we will conduct the review following the PRISMA statement guidelines. Any changes in the full review process will be recorded as appropriate.

### 2.2. Inclusion Criteria for Study Selection

#### 2.2.1. Study Type

We will include published randomized controlled trials (RCTs) focused on Tai Chi for CPMP. Language restrictions will be papers published in English and Chinese.

#### 2.2.2. Participants

CPMP is one subtype of chronic primary pain (CPP). According to the International Classification of Diseases, 11th Revision (ICD-11) [36], CPP is established as pain persisting for more than 3 months, associated with noteworthy emotional distress, as well as there is no other diagnosis better accounted for chronic pain. CPMP is chosen when CPP is located in the joints, muscles, bones, or tendons, including chronic primary neck pain, chronic primary thoracic pain, chronic primary low back pain, and chronic primary limb pain. Included studies will comply with established diagnostic criteria for chronic pain (e.g., ICD-10), which means the syndromes referring to neck pain, thoracic pain, low back pain, or limb pain that persists for more than 3 months, with or without additional provided information regarding the emotional disability, will be included. Studies that assess chronic secondary musculoskeletal pain (e.g., rheumatoid arthritis and osteoarthritis) will be excluded. In addition, we will only include RCTs involving patients aged 18 years or older (adults). There will be no limitations by sex, country, or region.

#### 2.2.3. Intervention

We will only include RCTs that compared a treatment group practicing Tai Chi as the sole approach with a control group with usual care, no treatment, or other forms of exercise or therapies (e.g., pharmacological, physiological, or educational interventions). Studies will not be included when Tai Chi participated in the control group as an adjunctive therapy.

#### 2.2.4. Outcomes

Reduction in pain intensity will be explored as the primary outcome as measured by the visual analog scale or numerical rating scale. Hospital Anxiety and Depression Scale scores, SF-36 Health Survey scores, Pittsburgh Sleep Quality Index scores, and adverse effects will be explored as secondary outcomes.

### 2.3. Search Methods to Identify Studies

#### 2.3.1. Electronic Searches

We will search relevant databases from inception to May 31, 2021: Cochrane Central Register of Controlled Trials, PubMed, EMBASE, China Knowledge Resource Integrated Database, the Chongqing VIP database, the Wanfang database, and China Biology Medicine Disc.

#### 2.3.2. Searching Other Resources

We will search clinical trial databases such as the World Health Organization International Clinical Trials Registry Platform (WHO ICTRP) (http://apps.who.int/trialsearch/) and ClinicalTrials.gov (http://clinicaltrials.gov/) for more data. We will browse reference material of retrieved clinical trials and review articles to find potentially relevant data. In addition, we will also search gray literature databases (OpenGrey, GreyNet, and GreyLit).

#### 2.3.3. Search Strategy

The search terms will include Tai Chi (e.g., “Tai Chi” or “Tai Ji” or “Tai-ji” or “T'ai Chi” or “Tai Chi Quan” or “Tai Ji Quan” or “Tai Chi Chuan”), chronic primary musculoskeletal pain (e.g., “pain” or “chronic pain” or “pain management” or “musculoskeletal pain” or “physical suffering” or “neck pain” or “neck ache” or “cervical pain” or “cervicalgia” or “cervicodynia” or “neckache” or “thoracic pain” or “limb pain” or “arm pain” or “leg pain” or “low back pain” or “lumbago” or “lower back pain” or “low back ache” or “low backache”), and randomized controlled trial (e.g., “randomized controlled trial” or “clinical trial” or “comparative study” or “controlled clinical trial”). See Text.  1 in the Supplementary Material for the primary search strategy, and this strategy will be revised appropriately in the process for other databases.

### 2.4. Analysis

#### 2.4.1. Study Selection

Hits returned by the electronic searches will be imported into Endnote X9 by two reviewers (authors) independently. After deleting duplicates, the study titles and abstracts will be screened by two independent reviewers. The full-text articles that appear to meet the inclusion criteria will be accessed to confirm whether they are eligible for inclusion in our review according to the predefined criteria. Any disagreement regarding study selection will be resolved by consensus. If the two reviewers cannot reach consensus, a third reviewer will make the final decision.

#### 2.4.2. Data Extraction and Management

Two reviewers will extract the data from all included articles using a standardized form to assess study quality and for evidence synthesis. The extracted data will include the first author, publication year, country, study design and characteristics, risk-of-bias items, and PICOTS (i.e., population, intervention, comparator, outcome, timing, and setting). Where consensus on data extraction cannot be reached through negotiation, a third reviewer will make the final judgment. When necessary and feasible, the corresponding authors of the selected studies will be contacted to obtain missing or incomplete data.

#### 2.4.3. Assessment of Reporting Quality and Risk of Bias

The risk of bias of each included study will be independently evaluated by two reviewers using an established risk of bias tool. The risk of bias tool will be drawn from the most recent Cochrane Handbook for Systematic Reviews of Interventions [37]. This assessment will cover (1) concealment of allocation; (2) blinding of participants, manipulators, and outcome appraiser; (3) generation of random sequence; (4) selective reporting outcomes; (5) incomplete outcome data; and (6) other biases. We will categorize the risk of bias into three levels: unclear risk, low risk, or high risk. Any differences in decisions will be resolved by consensus; if the two reviewers cannot reach agreement, a third reviewer will make the final decision.

#### 2.4.4. Measures of Treatment Effect

For continuous data, the mean difference (MD) and 95% confidence interval (CI) will be used to evaluate the effect of Tai Chi. When results are measured on different outcome scales, we will use the standardized MD with 95% CI for the analysis. We will use risk ratio with 95% CI to evaluate the effect of the treatment for dichotomous data.

#### 2.4.5. Assessment of Publication Bias

If this systematic review includes 10 or more articles, funnel plots will be used to test the risk of publication bias.

#### 2.4.6. Heterogeneity Analysis

Visual inspection of forest plots, the *I*^2^ statistic, and *χ*^2^ tests will be used to identify heterogeneity in the data. A random-effect model will be used when the included studies show statistically significant heterogeneity (*I*^2^ statistic >50% or *P* value <0.10). Otherwise, a fixed-effect model will be adopted.

#### 2.4.7. Data Synthesis

A quantitative synthesis will be conducted for outcomes reported in more than one homogeneous RCT. The systematic review will be performed using RevMan 5 software (version 5.3; Copenhagen: The Nordic Cochrane Centre, The Cochrane Collaboration, 2014). We will choose random- or fixed-effect models based on the analysis of heterogeneity. Randomized individuals will be considered in unit-of-analysis issues. If a meta-analysis is not appropriate because of clinical/methodological issues or statistical heterogeneity, a narrative summary of the findings or relevant subgroup analyses will be used. We will also provide an overall summary of outcomes in CPMP, with this summary categorized by population type (type of CPMP).

#### 2.4.8. Subgroup Analyses

When sufficient data are available, subgroup analyses will be conducted based on different types of CPMP (i.e., chronic primary neck pain, chronic primary thoracic pain, chronic primary low back pain, or chronic primary limb pain).

#### 2.4.9. Sensitivity Analysis

By assessing the criteria including sample size, heterogeneity, and statistical model (random- or fixed-effect model), a sensitivity analysis will be conducted to identify the stability of our conclusions.

#### 2.4.10. Grading the Quality of Evidence

In the final report, two reviewers will present findings concerning the quality of evidence by independently assessing the evidence quality for outcomes using the Grading of Recommendations Assessment approach [38]. This assessment of evidence quality includes risk of bias, heterogeneity, indirectness, imprecision, and publication bias. The quality of the evidence will be classified as high, moderate, low, or very low.

## 3. Discussion

Treatment for CPMP remains unsatisfactory despite a significant increase in the number of clinical RCTs focused on CPMP. It has been noted that, as a therapy for CPMP, Tai Chi has the advantages of few side effects, low cost, and that it can be widely used by the general population. Traditional Chinese medicine theories indicate that Tai Chi can dredge meridians and collaterals and regulate the circulation of Qi and blood [39]. In addition, when performed as aerobic training, Tai Chi may be conductive to improving musculoskeletal fitness, thereby increasing the physical performance and augmenting pain tolerance [40], dampening pain perception, and reducing pain sensitivity [41]. Clinical studies have shown that Tai Chi can relieve pain and ameliorate musculoskeletal conditions, along with having beneficial effects on psychological well-being. According to our preliminary search, no systematic review is available that includes trials evaluating the clinical effect and safety of Tai Chi for patients suffering from CPMP. We aim to clarify the effect and safety of Tai Chi for CPMP by summarizing available evidence. Our findings are also expected to identify literature disparities and provide clinicians with direction for treatments of CPMP. If Tai Chi effectively and safely improves CPMP, it is likely to be a preferable alternative or adjunct therapy to pharmacological/surgical treatment for CPMP.

## Data Availability

The data and materials are available from the corresponding authors upon request.

## Abbreviations

CPMP: Chronic primary musculoskeletal pain
CMP: Chronic musculoskeletal pain
CPP: Chronic primary pain
PRISMA-P: Preferred Reporting Items for Systematic Reviews and Meta-Analysis Protocols
ICD: International Classification of Diseases
RCT: Randomized controlled trial
MD: Mean difference
CI: Confidence interval.

## Supplementary Materials

Supplementary Materials S1: PRISMA 2009 checklist. S2: Text. 1: example search strategy (PubMed).

## References

[1] Monti S., Caporali R. (2015). Chronic pain: the burden of disease and treatment innovations. *Reumatismo*.

[2] Jackson T., Thomas S., Stabile V., Shotwell M., Han X., McQueen K. (2016). A systematic review and meta-analysis of the global burden of chronic pain without clear etiology in low- and middle-income countries. *Anesthesia & Analgesia*.

[3] GBD 2017 Disease and Injury Incidence and Prevalence Collaborators (2018). Global, regional, and national incidence, prevalence, and years lived with disability for 354 diseases and injuries for 195 countries and territories, 1990–2017: a systematic analysis for the global burden of disease study 2017. *Lancet*.

[4] Yongjun Z., Tingjie Z., Xiaoqiu Y. (2020). A survey of chronic pain in China. *Libyan Journal of Medicine*.

[5] Clauw D. J., Essex M. N., Pitman V., Jones K. D. (2019). Reframing chronic pain as a disease, not a symptom: rationale and implications for pain management. *Postgraduate Medicine*.

[6] Cheatle M. D., Foster S., Pinkett A., Lesneski M., Qu D., Dhingra L. (2016). Assessing and managing sleep disturbance in patients with chronic pain. *Anesthesiology Clinics*.

[7] Choy E. H. S. (2015). The role of sleep in pain and fibromyalgia. *Nature Reviews Rheumatology*.

[8] Zhuo M. (2016). Neural mechanisms underlying anxiety-chronic pain interactions. *Trends in Neurosciences*.

[9] Dureja G. P., Jain P. N., Shetty N. (2014). Prevalence of chronic pain, impact on daily life, and treatment practices in India. *Pain Practice*.

[10] Mills S. E. E., Nicolson K. P., Smith B. H. (2019). Chronic pain: a review of its epidemiology and associated factors in population-based studies. *British Journal of Anaesthesia*.

[11] Yeh G. Y., Wood M. J., Lorell B. H. (2004). Effects of tai chi mind-body movement therapy on functional status and exercise capacity in patients with chronic heart failure: a randomized controlled trial. *The American Journal of Medicine*.

[12] Fogarty J. N., Murphy K. J., McFarlane B. (2016). Taoist tai chi and memory intervention for individuals with mild cognitive impairment. *Journal of Aging and Physical Activity*.

[13] Bicket M. C., Mao J. (2015). Chronic pain in older adults. *Anesthesiology Clinics*.

[14] Warner M., Chen L. H., Makuc D. M. (2009). Increase in fatal poisonings involving opioid analgesics in the United States, 1999–2006. *NCHS Data Brief*.

[15] Gomes T., Mamdani M. M., Dhalla I. A., Paterson J. M, Juurlink D. N (2011). Opioid dose and drug-related mortality in patients with nonmalignant pain. *Archives of Internal Medicine*.

[16] Polkey M. I., Qiu Z.-H., Zhou L. (2018). Tai chi and pulmonary rehabilitation compared for treatment-naive patients with COPD. *Chest*.

[17] Niu R., He R., Luo B.-l., Hu C. (2014). The effect of tai chi on chronic obstructive pulmonary disease: a pilot randomised study of lung function, exercise capacity and diaphragm strength. *Heart, Lung and Circulation*.

[18] Barrow D. E., Bedford A., Ives G., O’Toole L., Channer K. S. (2007). An evaluation of the effects of Tai Chi Chuan and Chi Kung training in patients with symptomatic heart failure: a randomised controlled pilot study. *Postgraduate Medical Journal*.

[19] Lam L. C., Chan W. M., Kwok T. C., Chiu H. F (2014). Effectiveness of Tai Chi in maintenance of cognitive and functional abilities in mild cognitive impairment: a randomised controlled trial. *Hong Kong Medical Journal = Xianggang Yi Xue Za Zhi*.

[20] Li F., Harmer P., Liu Y., Chou L.-S. (2014). Tai Ji Quan and global cognitive function in older adults with cognitive impairment: a pilot study. *Archives of Gerontology and Geriatrics*.

[21] Irwin M. R., Olmstead R., Breen E. C. (2015). Cognitive behavioral therapy and tai chi reverse cellular and genomic markers of inflammation in late-life insomnia: a randomized controlled trial. *Biological Psychiatry*.

[22] Irwin M. R., Olmstead R., Carrillo C. (2014). Cognitive behavioral therapy vs. Tai Chi for late life insomnia and inflammatory risk: a randomized controlled comparative efficacy trial. *Sleep*.

[23] Marley J., Tully M. A., Porter-Armstrong A., Bunting B., O’Hanlon J., McDonough S. M. (2014). A systematic review of interventions aimed at increasing physical activity in adults with chronic musculoskeletal pain-protocol. *Systematic Reviews*.

[24] Huston P., McFarlane B. (2016). Health benefits of tai chi: what is the evidence?. *Can Fam Physician*.

[25] Wang C., Schmid C. H., Hibberd P. L. (2009). Tai Chi is effective in treating knee osteoarthritis: a randomized controlled trial. *Arthritis & Rheumatism*.

[26] You T., Ogawa E. F., Thapa S. (2018). Tai Chi for older adults with chronic multisite pain: a randomized controlled pilot study. *Aging Clinical and Experimental Research*.

[27] Lauche R., Stumpe C., Fehr J. (2016). The effects of tai chi and neck exercises in the treatment of chronic nonspecific neck pain: a randomized controlled trial. *The Journal of Pain*.

[28] Allen J., Imbert I., Havelin J. (2017). Effects of treadmill exercise on advanced osteoarthritis pain in rats. *Arthritis & Rheumatology*.

[29] Stagg N. J., Mata H. P., Ibrahim M. M. (2011). Regular exercise reverses sensory hypersensitivity in a rat neuropathic pain model. *Anesthesiology*.

[30] Zou L., Zhang Y., Liu Y. (2019). The effects of tai chi chuan versus core stability training on lower-limb neuromuscular function in aging individuals with non-specific chronic lower back pain. *Medicina*.

[31] Hall A. M., Maher C. G., Lam P., Ferreira M., Latimer J. (2011). Tai chi exercise for treatment of pain and disability in people with persistent low back pain: a randomized controlled trial. *Arthritis Care & Research*.

[32] Peng P. W. H. (2012). Tai chi and chronic pain. *Regional Anesthesia and Pain Medicine*.

[33] Wang F., Lee E.-K. O., Wu T. (2014). The effects of tai chi on depression, anxiety, and psychological well-being: a systematic review and meta-analysis. *International Journal of Behavioral Medicine*.

[34] Hall A., Maher C., Latimer J., Ferreira M. (2009). The effectiveness of tai chi for chronic musculoskeletal pain conditions: a systematic review and meta-analysis. *Arthritis & Rheumatism*.

[35] Hall A., Copsey B., Richmond H. (2017). Effectiveness of tai chi for chronic musculoskeletal pain conditions: updated systematic review and meta-analysis. *Physical Therapy*.

[36] Nicholas M., Vlaeyen J. W. S., Rief W. (2019). The IASP classification of chronic pain for ICD-11: chronic primary pain. *Pain*.

[37] Higgins J. P. T., Altman D. G., Gotzsche P. C. (2011). The cochrane collaboration’s tool for assessing risk of bias in randomised trials. *BMJ*.

[38] Guyatt G. H., Oxman A. D., Vist G. E. (2008). GRADE: an emerging consensus on rating quality of evidence and strength of recommendations. *BMJ*.

[39] Wang C., Schmid C. H., Fielding R. A. (2018). Effect of tai chi versus aerobic exercise for fibromyalgia: comparative effectiveness randomized controlled trial. *BMJ*.

[40] Soriano-Maldonado A., Ortega F. B., Munguía-Izquierdo D. (2015). Association of cardiorespiratory fitness with pressure pain sensitivity and clinical pain in women with fibromyalgia. *Rheumatology International*.

[41] Naugle K. M., Fillingim R. B., Riley J. L. (2012). A meta-analytic review of the hypoalgesic effects of exercise. *The Journal of Pain*.

