# Supplementary material for: Tai Chi for Improving Chronic Primary Musculoskeletal Pain: Protocol for a Systematic Review
Source: Evid Based Complement Alternat Med. 2021 Jun 21;2021:9932336. doi: 10.1155/2021/9932336 (PMC8238584; doi:10.1155/2021/9932336)
Supplement: Supplementary Materials — S1: PRISMA 2009 checklist. S2: Text. 1: example search strategy (PubMed). [file 9932336.f1.zip › 9932336.f1/S2 Text.1 Example Search Strategy (Pubmed).docx]

Text.1 Example Search Strategy (Pubmed)

#1 (Tai Ji[Mesh]) OR (Tai Ji[tiab]) OR (Tai Chi[tiab]) OR (Tai-ji[tiab]) OR (Chi, Tai[tiab]) OR (Tai Ji Quan[tiab]) OR (Ji Quan, Tai[tiab]) OR (Quan, Tai Ji[tiab]) OR (Taiji[tiab]) OR (Taijiquan[tiab]) OR (T'ai Chi[tiab]) OR (Tai Chi Chuan [tiab]) OR (Tai Chi*stick) OR (Tai Chi*staff)

#2 (pain[Mesh]) OR (pain[tiab]) OR (chronic pain[Mesh]) OR (chronic Pain[tiab]) OR (pain management[Mesh]) OR (pain management[tiab]) OR (musculoskeletal pain[Mesh]) OR (musculoskeletal pain[tiab]) OR (physical suffering[tiab]) OR (neck pain[Mesh]) OR (neck pain[tiab]) OR (neck ache[tiab]) OR (cervicalgia[tiab]) OR (cervicodynia[tiab]) OR (neckache[tiab]) OR (cervical pain[tiab]) OR (low back pain[Mesh]) OR (low back pain[tiab]) OR (lumbago[tiab]) OR (lower back pain[tiab]) OR (low back ache [tiab]) OR (low backache[tiab]) OR (thoracic pain[tiab]) OR (limb pain[Mesh]) OR (limb pain[tiab]) OR (arm pain[tiab]) OR (leg pain[tiab])

#3 (human [Mesh]) AND ((clinical trial[pt]) OR (meta-analysis[pt]) OR (randomized controlled trial[pt]) OR (randomized controlled trial [Mesh]) OR (comparative study[pt]) OR (controlled clinical trial[pt]))

#4 #1 and #2

#5 #3 and #4
